# Cellular angiofibroma in women: a review of the literature

**DOI:** 10.1186/s13000-015-0361-6

**Published:** 2015-07-19

**Authors:** Vincenzo Dario Mandato, Susanna Santagni, Alberto Cavazza, Lorenzo Aguzzoli, Martino Abrate, Giovanni Battista La Sala

**Affiliations:** Unit of Obstetrics and Gynecology, IRCCS-Arcispedale S. Maria Nuova di Reggio Emilia, Viale Risorgimento n 80, Reggio Emilia, Italy; Unit of Pathology, IRCCS-Arcispedale S. Maria Nuova di Reggio Emilia, Reggio Emilia, Italy; Unit of Obstetrics and Gynecology, University of Modena e Reggio Emilia, Reggio Emilia, Italy

**Keywords:** Cellular angiofibroma, Vulvovaginal soft tissue tumour, Mesenchymal tumour, Vulvovaginal benign tumour

## Abstract

Cellular Angiofibroma (CA) represents a quite recently described mesenchymal tumour that occurs in both genders, in particular in the vulvo-vaginal region in women and in the inguino-scrotal area in men. The first description of this tumour dates from Nucci et al. article in 1997; since then, the literature reports different reviews and case report of this tumour in both genders, but no article specifically addressing CA treatment and follow-up in women. In this review we collected all 79 published female CA cases, analyzing the clinical, pathological and immunohistochemical features of the tumour.

CA affects women mostly during the fifth decade of life, it is generally a small and asymptomatic mass that mainly arises in the vulvo-vaginal region, although there are reported pelvic and extra-pelvic cases. The treatment requires a simple local excision due to an extremely low ability to recurrent locally and no chance to metastasize. Throughout the immunohistochemical and pathological findings it is also easily possible a differential diagnosis from the other soft tissue tumours which affect the vulvo-vaginal area, such as spindle cell lipoma, solitary fibrous tumour, angiomyofibroblastoma and aggressive angiomyxoma.

## Introduction

Cellular angiofibroma (CA) belongs to soft tissue tumours that predominantly occur in the distal genital tract of both genders: vulvo-vaginal region in women and inguino-scrotal area in men [1, 2], although extragenital localizations have also been described [3–5].

The first soft tissue tumour with a relatively pelvic site-specificity was a benign stromal polyp described in the early 1960 [6].

**Fig. 1:**
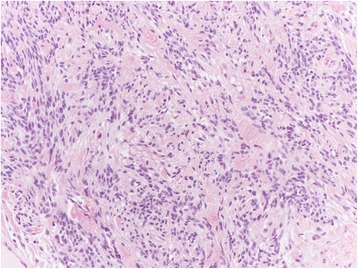
Leiomyoma at hematoxylin-eosin, 200X

**Fig. 2:**
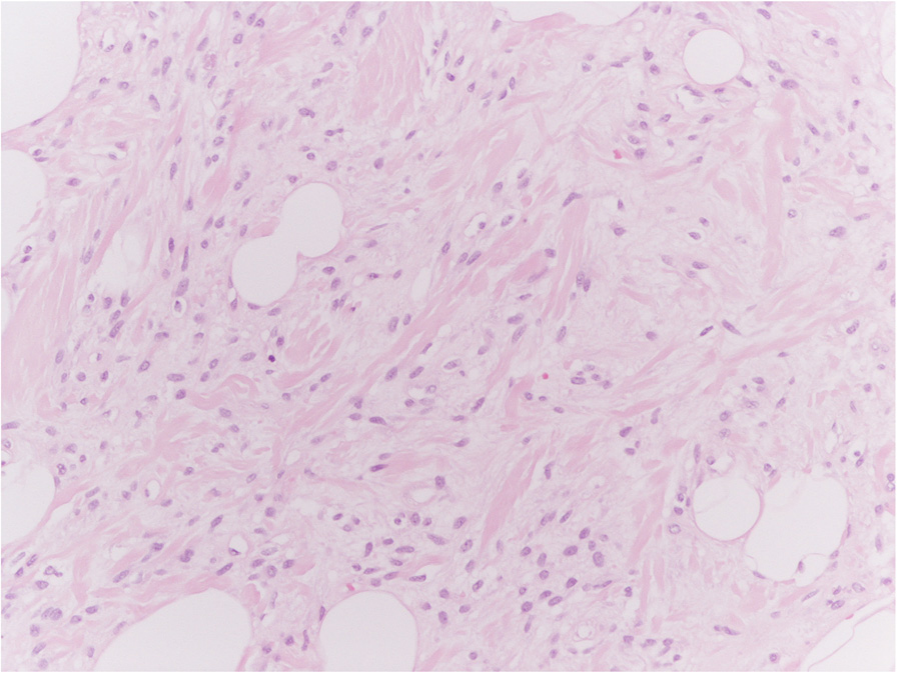
Lipoma at hematoxylin-eosin, 200X

**Fig. 3:**
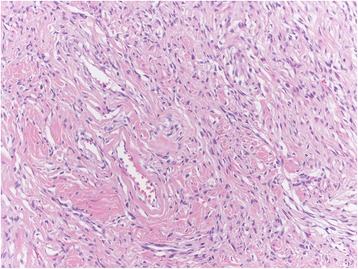
Solitary fibrous at hematoxylin-eosin, 200X

**Fig. 4:**
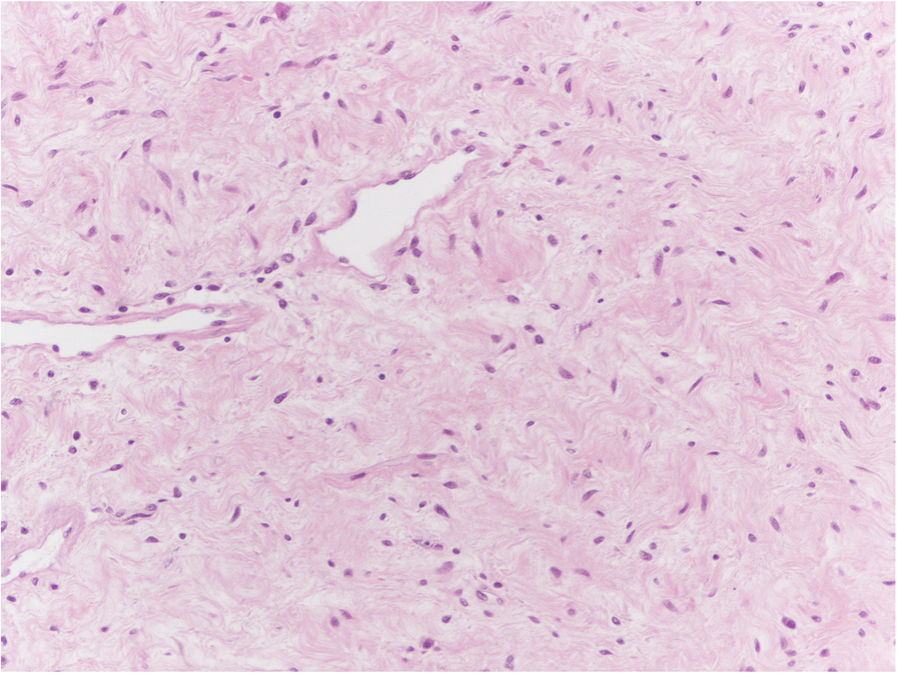
Cellular angiofibroma at hematoxylin-eosin, 200X

**Fig. 5:**
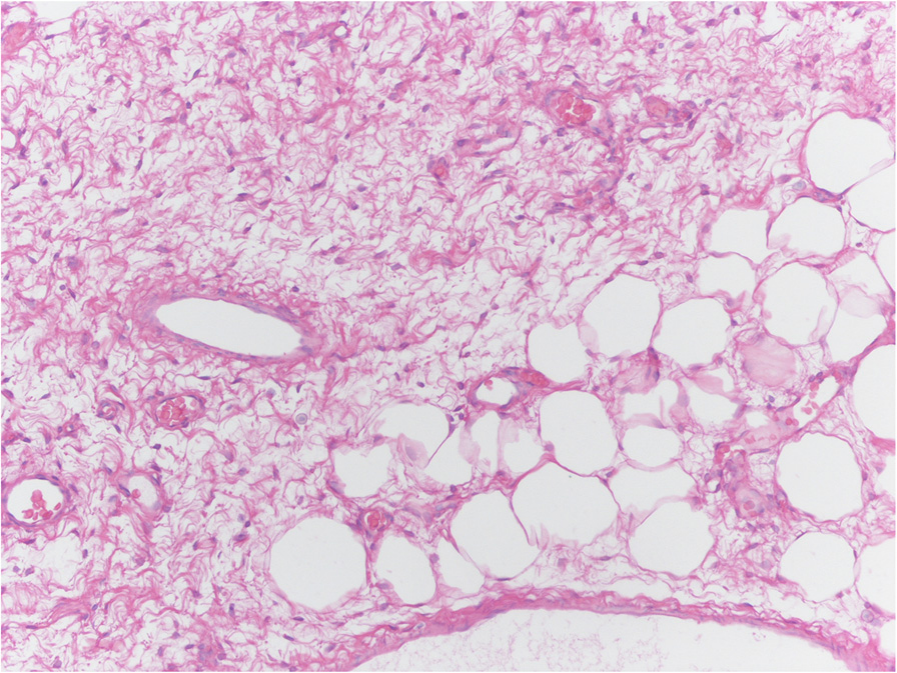
Aggressive angiomyxoma at hematoxylin-eosin, 200X

**Fig. 6:**
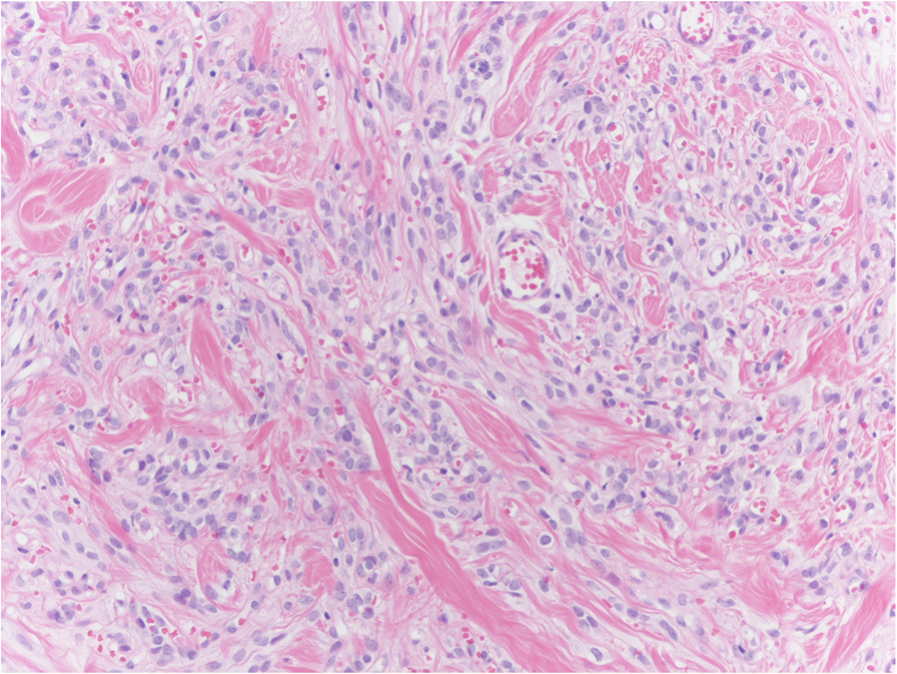
Angiomyofibroblastoma at hematoxylin-eosin, 200X

Among the mesenchymal tumours involving the vulva, we can distinguish the lesions that can arise at any site, including the vulva, and those more characteristics of this area. The former group includes leiomyoma (Fig. 1), lipoma (Fig. 2) and solitary fibrous tumour (Fig. 3) while the second group includes CA (Fig. 4) along with aggressive angiomyxoma (Fig. 5), angiomyofibroblastoma (Fig. 6) and fibroepithelial stromal polyp [7].

Cellular angiofibroma is a rare benign mesenchymal lesion, first described by Nucci et al. in 1997 [1] in a series of 6 cases that occurred almost exclusively in the vulva of middle-aged women. A short time later, Laskin et al. [2] described 11 cases of a histologically similar lesion named “angiomyofibroblastoma-like” tumour, which affects the adult men in the inguino-scrotal area. The World Health Organization classification [8] established that the term “Cellular angiofibroma” includes this kind of lesion in both females and males, because there are no reproducible morphologic differences between the two genders. Macroscopically these lesions are commonly well circumscribed, localized in the superficial soft tissue and are characterized by 2 main components: bland spindle cells and small to medium-sized vessels with mural hyalinization [9].

Since the first description by Nucci, only few studies have been published in the literature, most of which consisted of single case-reports or reviews which include cases from both genders.

Here, we reviewed all the cases of CA arising in women since 1997, to clarify the outcome and to identify the best treatment.

## Review

### Materials and methods

We collected and analyzed the published articles in the literature regarding CA from January 1997 to December 2014, using Pubmed research and the terminologies “cellular angiofibroma”, “vulvovaginal mesenchymal tumour”, “vulvovaginal benign tumour”, “vulvovaginal stromal tumour” and “vulvovaginal soft tissue tumour”. We selected only the cases involving the female gender: a total of 79 published cases of CA were found. Where possible, we contacted the authors to update the follow-up data, in particular regarding local recurrences and/or metastases.

In the Tables 1 and 2 are reported the main clinical features of the 74 vulvo-vaginal and pelvic CA and the clinical finding of the 6 extra-pelvic CA, respectively.

**Table 1:** Clinical features of 74 cases of Cellular Angiofibroma in vulvo-vaginal and pelvic regions

Case	Age	Author	Site	Size (media, cm)	Treatment	Follow-up (Months)	Recurrence
1	50	Nucci	Vulva	1,2	Simple excision	UK	UK
2	46	Nucci	Lab maj	2	Local excision + 2 reexcisions	19	NER
3	39	Nucci	Lab	2,15	Simple excision	12	NER
4	49	Nucci	Lab	1,2	Simple excision	UK	UK
5	43	Nucci	Vulva	2,3	UK	UK	UK
6	41	Nucci	perineum	3	UK	UK	UK
7	37	Colombat	Lab	2,5	UK	UK	UK
8	37	Curry	Clitoral Hood	3,5	UK	15	UK
9	53	Dufau	Lab maj	3,3	UK	UK	UK
10	46	Dargent	Lab maj	2,33	Simple excision	149	NER
11	49	Dargent	Clitoris	2,66	Simple excision	161	NER
12	49	Iwasa	Lab maj	2	Simple excision	5	UK
13	39	Iwasa	Vulva	/	UK	UK	UK
14	46	Iwasa	Lab maj	2	Simple excision	168	NER
15	50	Iwasa	Vulva	1,5	Simple excision	UK	UK
16	42	Iwasa	Vulva	3	Simple excision	75	NER
17	42	Iwasa	Perineum	/	UK	UK	UK
18	75	Iwasa	Vulva	2,8	Simple excision	59	Died for Breast cancer
19	27	Iwasa	Lab maj	1,1	Local excision + reexcision	50	NER
20	41	Iwasa	Vulva	2,7	Simple excision	54	NER
21	68	Iwasa	Vulva	/	Simple excision	17	NER
22	59	Iwasa	Lab maj	2	Simple excision	41	NER
23	49	Iwasa	Vulva	/	UK	UK	UK
24	37	Iwasa	Hymen	4,8	Local excision with positive margins	24	NER
25	38	Iwasa	vagina	/	UK	UK	UK
26	46	Iwasa	Vulva	2,9	Simple excision	35	NER
27	47	Iwasa	Lab maj	1,4	Simple excision	44	NER
28	22	Iwasa	Inguinal region	12	Local excision + reexcision	18	NER
29	52	Iwasa	Urethra	/	UK	UK	UK
30	47	Iwasa	Vulva	/	UK	UK	UK
31	48	Iwasa	Lab maj	3	Simple excision	8	NER
32	24	Iwasa	Vagina	11	UK	6	NER
33	31	Iwasa	Perineum	4	Simple excision	UK	UK
34	58	Iwasa	Vagina	0,6	Simple excision	UK	UK
35	50	Iwasa	Vulva	1,8	Simple excision	6	NER
36	58	Iwasa	Vulva	3	Simple excision	9	NER
37	50	Iwasa	Vulva	/	UK	UK	UK
38	49	McCluccage	Lab maj	4	Simple excision	6	Local recurrence
39	20	McCluccage	Not specified	2,4	Simple excision	240	NER
40	65	McCluccage	Lab maj	5	Simple excision	12	NER
41	59	McCluccage	Vulva	2	Simple excision	18	NER
42	58	Chen	Vulva	2,7	Piecemeal excision	75	NER
43	52	Chen	Vulva	3	Simple excision	27	NER, died for cancer of UK origin
44	34	Chen	Vulva	1,2	Simple excision	UK	UK
45	32	Chen	Vulva	4,85	Simple excision	UK	UK
46	25	Chen	Vulva	1,3	Simple excision	42	NER
47	43	Chen	Vulva	2,5	Simple excision	2	NER
48	59	Chen	Vulva	1,3	Simple excision	14	NER
49	46	Chen	Vulva	6,5	Simple excision	4	NER
50	71	Chen	Vulva	7,5	Simple excision	UK	UK
51	39	Chen	Vulva	/	Simple excision	7	NER
52	46	Chen	Vulva	2	Simple excision	UK	UK
53	41	Flucke	Perineum	3	Simple excision	UK	UK
54	39	Flucke	Vagina	1	Local excision with positive margins	75	NER
55	50	Flucke	Vulva	3	Local excision + reexcision	55	NER
56	51	Flucke	Lab maj	2,7	Marginal excision	66	NER
57	44	Flucke	Lab maj	2,3	Simple excision	UK	UK
58	50	Flucke	Vulva	4	Local excision with positive margins	UK	UK
59	48	Flucke	Vulva	8,5	Simple excision	UK	UK
60	42	Flucke	Vulva	2,2	Simple excision	UK	UK
61	63	Flucke	Clitoris	2,5	Local excision with positive margins	38	NER
62	27	Flucke	Lab maj	8	Marginal excision	UK	UK
63	42	Flucke	Vulva	1,7	Simple excision	30	UK
64	46	Flucke	Lab maj	3	Marginal excision	UK	UK
65	55	Flucke	Vulva	2,3	Simple excision	12	NER
66	57	Flucke	Vulva	4,5	Simple excision	6	NER
67	47	Flucke	Vulva	1,5	Local excision with positive margins	UK	UK
68	39	Flucke	Vagina	9	Marginal excision	UK	UK
69	51	Rua Micheletti	Lab maj	4,6	Simple excision	112	NER
70	31	Kerkuta	Lab maj	4	Simple excision	10	NER
71	77	Lane	Lab	4	Simple excision	12	NER
72	26	Arsenovic	Vulva	8,5	Simple excision	90	NER
73	55	Maggiani	Vagina	12,3	UK	UK	UK
74	20	Ahmadnia	Both lab maj	/	Simple excision	12	NER

*NER* not evidence of recurrence, *UK* unknown

**Table 2:** Clinical features of 6 cases of extra-pelvic Cellular Angiofibroma

Case	Age	Author	Site	Size (media, cm)	Treatment	Follow-up (Months)	Recurrence
75	52	Chen	Left hip	3,5	Local excision + reexcision	UK	UK
76	63	Flucke	Knee lateral	4	Simple excision	45	NER
77	43	Val-Bernal	Chest wall	7	Simple excision	203	NER
78	38	Val-Bernal	Left hypocondrium	3,5	Simple excision	104	NER
79	60	Mandato	Retroperitoneim pelvicc	3,9	Simple excision	6	NER
80	20	Ahmadnia	Left axilla + both breast	/	Simple excision	12	NER

*NER* not evidence of recurrence, *UK* unknown

### Results

#### Clinical features

The 79 affected women have an age at presentation ranging from 20 to 77 years (mean 46.1 years). The most common anatomic site is the vulvo-vaginal region, in particular labium majus area and vulva overall (18 and 35 cases, respectively). Outside the vulvo-vaginal region, 5 pelvic localizations are reported, including 4 perineum and an urethral site. Six extra-pelvic CA are reported, such as a left hip, lateral knee, chest wall, left axilla and breasts [10], left hypocondrium [3, 5] and more recently, we have reported the first case of retroperitoneal CA arising in right paravesical space of a post-menopausal woman [11].

The tumour size ranges from 0.6 to 12.3 cm, with a mean size of 3.6 cm. A clinical diagnosis before surgery is reported only in 25 cases. Among these, the most common clinical presentation resembles a Bartholin’s cyst (12/25, 48 % of cases), a not-specific solid mass (7/25, 28 % of cases), vulval cyst (3/25, 12 % of cases), leyomioma (2/12, 8 % of cases) and lipoma (1/25, 4 % of cases). In 8 cases of CA the time occurred between the tumour’s occurrence and the surgical treatment is reported: the mean is 16.6 month, ranging from 2 to 36 months.

CA is usually treated by simple excision; the involvement or not of the surgical margins is reported in 47/78 (60.2 %) CA with 18 positive surgical margins (18/47, 38.2 %) and 29 negative surgical margins (29/47, 61.7 %); only 5 cases (5/18, 27.8 %) of re-excision because involved the surgical margin are reported.

Follow-up data are reported in 48/79 (60.7 %) cases with a range of 3–240 months (mean 46.6 months): the available follow-up data show that CA tends to not develop local recurrences or metastasis also in case of atypia or sarcomatous transformation. In 7 of the 12 patients with atypia or sarcomatous transformation follow up data were available, no recurrence or metastases were reported after a median follow-up of 14 months [9].

One patients died of metastatic carcinoma of unknown primary origin, after 27 months from the diagnosis of CA with sarcomatous component. In an another one, the women died of breast cancer, 59 months after the CA diagnosis, without CA recurrence or metastases found meanwhile.

Only a local recurrence of vulvar CA is described after 6 months follow-up [12]. CA has been initially excised with a rim of free-tumour tissue [12].

#### Pathological features

A macroscopic/microscopic description was reported in 63/79 (79.7 %) CA. Grossly, the neoplasm is described as white or yellowish nodules, mostly firm and partly gelatinous of cystic in appearance, with a cut surface white tan to grayish in color. Macroscopically, three cases is recorded as polypoid lesions [13] and in other few cases is appreciated a multilobulated appearance. CA is usually well-circumscribed, although focal extension into surrounding soft tissue can be seen. Most lesions were found in superficial soft tissue while 5 cases, all in the vulvovaginal region, involve dermis [3]. There is reported only one case with foci of haemorrhage [3] and none showing foci of necrosis.

Microscopically, the CA is a cellular neoplasm, composed of bland spindle shaped cells, proliferating in an edematous to fibrous stroma, containing wispy collagen bundles, numerous small to medium-sized thick-walled, often hyalinized, vessels and a minor component of adipose tissue [7]. The spindle cell component in most cases is quite cellular and randomly distributed throughout the lesion, focally forming short fascicles but generally without any particular pattern [3]. The spindle cells usually have short, fusiform, ovoid, sometimes tapering or polygonal nuclei with incospicuous nucleoli, while their cytoplasm is palely eosinophilic with indistinct, ill-defined borders or bipolar dendritic processes. Nuclear grooves and intranuclear inclusions are commonly observed [13]. A moderate number of inflammatory cells, comprising lymphocytes and rarely neutrophils, are scattered throughout the stroma [3]; the mast cells are often abundant while multinucleated giant cells or epithelioid cells are absent in this neoplasm.

Mitotic activity is very variable: indeed, a prominent mitotic activity may be occasionally observed, and abnormal mitoses and cellular atypia are rarely present.

CA with atypia or sarcomatous trasformation was reported in 12 women [9], no necrosis or hemorrhage was observed in any case and the atypical or sarcomatous component wasn’t morphologically recognizable on macroscopically examination. Microscopically, the atypical changes ranged from severely atypical cells disseminated within a background of usual CA to the formation of a discrete atypical foci. Similarly, the sarcomatous component showed variable features, including an atypical lipomatous tumour-like component, pleomorphic liposarcoma and pleomorphic sarcoma [9].

#### Immunohistochemical features

In the Table 3 are summarized the available data about the immunohistochemical findings of the selected articles. Immunohistochemical findings were reported only in 44/79 (55.7 %) published CA because many articles don’t distinguish the immunohistochemical features between the male and female tumours.

**Table 3:** Immunohistochemical features of cellular angiofibroma in women reported in literature

Case	Author	Vimentin	CD34	S-100	α-SMA	Desmin	Keratin	EMA	ER	PR	h-Caldesmon	CD10	CD99	CD31	CD117
1	Nucci	+	-	-	-	-	-	-							
2	Nucci	+	-	-	-	-	-	-							
3	Nucci	+	-	-	-	-	-	-							
4	Nucci	+	-	-	-	-	-	-							
5	Nucci		+												
6	Nucci		+												
10	Dargent	+	+	-	+	-		-	+	+	-		+	-	-
11	Dargent	+		-				-	+	+	-		+	-	-
12	Iwasa		+	-	+	+			+	+					
13	Iwasa		+	-	+	+			+	+					
14	Iwasa		+	-	+	-			+	+					
15	Iwasa		+	-	+	-			+	+					
16	Iwasa		+	-	-	-			+	+					
17	Iwasa		+	-	-	-			-	+					
18	Iwasa		+	-	-	-			-	+					
19	Iwasa		+	-	-	-			-	+					
20	Iwasa		+	-	-	-			-	+					
21	Iwasa		+	-	-	-			-	-					
22	Iwasa		+	-	-	-									
23	Iwasa		-	-	-	-									
24	Iwasa		-	-	-	-									
25	Iwasa		-	-	-	-									
26	Iwasa		-	-	-	-									
27	Iwasa		-	-	-	-									
28	Iwasa		-	-	-	-									
29	Iwasa		-	-	-	-									
30	Iwasa		-	-	-	-									
31	Iwasa		-	-	-	-									
32	Iwasa		-	-	-	-									
33	Iwasa		-	-	-	-									
34	Iwasa		-	-	-	-									
35	Iwasa		-	-	-	-									
38	McCluccage	+	-	-	-	-		+	+	+	-	-			
39	McCluccage	+	+	-	-	-		-	-						
40	McCluccage	+	+	-	-	-		-	+	+	-	+			
41	McCluccage	+	-	-	-	-		-	+	+	-	-			
69	Rua Micheletti	+	-	-	-	-									
70	Kerkuta	+	-	-	-	-			+	+					
71	Lane	+	+	-	-	-			+	+					
74	Arsenovic	+	+	-	-	-			+	+					
73	Maggiani		-	-	+	-			+	+					
76	Val-Bernal	+	+	-	-	-	-		-	+			+		
77	Val-Bernal	+	-		-		-	-							
78	Mandato		+						+						

*SMA* smooth muscle actin, *EMA* epithelial membrane antigen, *ER*/*PR* estrogen/progesterone receptor

Tumour cells are consistently negative for S-100protein (40/40 cases, 100 %), while they show positivity for CD34 in 45.4 % (20/44) of the cases. The CD34 positivity, together with typical muscle-markers positivity, as α-SMA and desmin, in an extremely minority of tumours (α-SMA positivity in 6 cases on 40 tested, 15 % and desmine positivity in 2/39 examined cases, 5.1 %), suggest a probable fibroblastic rather than myofibroblastic differentiation. When examined, the tumour cells also show an immunoreactivity for Vimentin. Another interesting finding is the expression of Estrogen receptor and/or Progesterone receptors by neoplastic cells [5, 14–16], which can suggests the role of an hormonal disturbance in the pathogenetic mechanisms of this tumour. When evaluated, all cases of CA show h-Caldesmon negativity (5/5 cases, 100 %) and Keratin negativity (6/6 cases, 100 %).

Multifocal or diffuse p16 expression is present in the atypical or sarcomatous areas of the CA, whilst is scattered or absent in usual CA [9]. In Table 4 are summarized the immunohistochemical findings in the other soft tissue tumors compared with CA [17, 18].

**Table 4:** Immunohistochemical features of CA and vulvar tumours for differential diagnosis

	Vimentin	CD34	S-100	α-SMA	Desmin	Keratin	EMA	ER	PR	h-Caldesmon	CD-10	ACTIN	STAT-6
Agressive Angiomyxoma	3+	1+		1+	3+			3+	3+			1+	
Angiomyfibroblastoma	3+	1+		3+	3+			3+	3+			3-/1+	
Ffibroepithelial stromal polyp	3+				3+			1+	1+			1+	
Solitary fibrous tumour		3+											3+
Smooth muscle neoplasm					3+							3+	
Spindle cell lipoma		3+											
Angiofibroma of soft tissue		1+		1+	1+		2+						
Superficial myofibroblastoma of the lower female genital tract	3+	1+	3-	1+	2+	3-	3-	3+	3+				
Cellular Angiofibroma	3+	2+	3-	2-	2-		3-	3+	3+	2-		3-	

(3+): most of cases positive; (2+): half of cases positive; (1+): few/some cases postive; (3-): typically negative; (2-): usually negative

### Discussion

Cellular angiofibroma is a quite recently described rare, benign stromal tumour that occurs equally among men and women: in female patients, CA most frequently arises in the vulvo-vaginal region, although there are described a lot of pelvic and extra-pelvic cases. Women are affected most often in the fifth decade (mean age 46.1 years), earlier than men, affected in the seventh decade. CA is characterized by its small size (mean value 3.6 cm) and usually well-circumscribed margins; it tends to be an asymptomatic and slowly enlarging mass, so CA tends to gradually increase in size after approximately 1–2 asymptomatic years, inducing women to check with their doctor long time after the tumour onset. CA is usually misdiagnosed with a Bartholin’s cyst (12/25, 48 % of cases), a not-specific solid mass (7/25, 28 % of cases), vulval cyst (3/25, 12 % of cases), leyomioma (2/12, 8 % of cases) and lipoma (1/25, 4 % of cases). Six extra-pelvic CA are reported in literature. Exceptionally, a young woman presented both vulvar and extra-pelvic CA, particularly two vulvar CA on the right and the left labia majora and three extravulvar CA on the left axilla and both breasts were reported [10].

Nevertheless exist exceptional early symptomatic forms, as well as our described case-report [11] in which the woman complained coxalgia due to tumour’s position, in retroperitoneal site, very near to obturator nerve.

There are many mesenchymal tumour which enter into the differential diagnosis with CA as spindle cell lipoma, solitary fibrous tumour, mammary-type myofibroblastoma, angiomyfibroblastoma, aggressive angiomyxoma and smooth muscle tumour (Table 4). Exceptionally, CA has been diagnosed with these mesenchymal tumours, such as with lipoma [19].

*Spindle cell lipoma* was first described by Enzinger and Harvey [20] in 1975 as a benign lipomatous tumour with a predilection for shoulder, back and posterior neck region of middle-aged to elderly men. Occasional cases may occur in a wide variety of sites, including one reported case arising in the vulva [21]. The tumour consists of mature adipocytes, bland spindle cells and short bundles of brightly eosinophilic ropy collagen [7]. In fact, CA and spindle cell lipoma have similar histological features, but can be distinguished by the rarity of spindle cell lipoma in vulvovaginal region and the presence, in CA, of more numerous vessels with thickened, hyalinized walls, compared with capillary-sized thin-walled vessels in spindle cell lipoma. The immunostaining is not helpful because the spindle cells of both tumours are CD34 positive, in particular CD34 is positive in about 60 % of CA and in 100 % of spindle cell lipoma [8].

*Solitary fibrous tumour* has been reported in a wide variety of locations, including vulva and perineum [22, 23]. This tumour shows a patternless spindle cell proliferation of alternating hypercellular and hypocellular areas, associated with dense collagen bundles, stromal keloidal-type hyalinization and hemangiopericytoma-like vessels [8]. Both solitary fibrous tumour and CA may contain fat in the lesion [24] and show often positivity for CD34 [8], but CA differs due to more evenly distributed cellularity, bland spindle cells with short bundles of collagen and more rounded small- to medium-sized vessels.

*Mammary-type myofibroblastoma,* as we know for its typical breast localization, has been described also at extramammary locations, especially in the inguinal region, with a male predominance [25]*.* Grossly, this lesion appears as a well circumscribed, nodular mass and, microscopically, it is composed of spindle cells and adipose tissue, separated by coarse bundles of hyalinized collagen. In comparison of CA, the vessels in mammary-type myofibroblastoma are incospicuous and the spindle cells have a more fascicular arrangement. The tumour cells consistently exhibit coexpression of desmin and CD34 [25].

All these described tumours share similar morphologic features and are characterized by bland ovoid to spindle-shaped cells with wispy collagen, variably sized thick-walled blood vessels and immunoreactivity to CD34.

In consideration of the clinical features, *aggressive angiomyxoma (AA)* and *angiomyofibroblastoma (AMF)* should also be included in the differential diagnosis of CA. The former is a typical locally recurring tumour of the vulvo-vaginal/perineal/inguinal regions, which occurs mainly in women in third to fifth decades [26–28]. It is easily distinguishable from CA because it is generally a large deep-seated lesion, poorly circumscribed with infiltrative edges: AA tends to display an infiltrative growth with entrapment of mucosal glands, fat, muscle and nerves. Microscopically, aggressive angiomyxoma is hypocellular, compared with CA, and it is composed of short spindle tumour cells with minimal atypia in myxoid stroma. Small clusters of smooth muscle cells surrounding or “spinning off from” blood vessels are a characteristic feature of AA [3]. There is a variable positivity for desmin, smooth muscle actin and CD34 [7, 27, 28].

*Angiomyofibroblastoma* is a benign tumour most commonly occurring in the vulva of peri- and post-menopausal women [29, 30]. Like CA, AMF is well circumscribed, even well demarcated, usually with a thin fibrous capsule but, unlike to CA, it is characterized by alternating hypocellular and hypercellular areas, together with multinucleate cells and epithelioid or plasmacytoid cells arranged in cords and nets around vessels. In fact, the characteristic feature of angiomyofibroblastoma is the perivascular accentuation of tumor cells [29, 30]. In aid to differential diagnosis, the immunochemistry is very useful because the tumour cells of AMF express desmin and rarely CD34 and smooth muscle actin [7, 29, 30].

Finally, vulvar *smooth muscle tumours* can be easily distinguished from CA because the latter lacks typical features of smooth muscle differentiation and it is usually desmin negative. The smooth muscle neoplasm are classically composed of cells with blunt-ended nuclei and eosinophilic cytoplasm, although these histological features could be absent when the tumour is localized in the vulvovaginal region.

The immunohistochemical features of CA may be helpful in differential diagnosis with the other vulvovaginal soft tissue tumours. An interesting immunohistochemical finding is the Estrogen and/or Progesterone receptors (ER/PR) expression by the CA. The occurrence of CA during menopausal transition and post-menopausal period and the description of a two cases associated with long-term estrogen replacement therapy [11, 15] support the hypothesis of an hormonal pathogenetic origin. However, the direct role of these steroids in the CA pathogenesis still remains unclear, since a subset of mesenchymal cells of the distal female genital tract normally expresses these receptors and, at the same time, the neoplastic cells in other soft tissue disorders, arising from the vulva, may also show immunoreactivity for ER and/or PR [31].

Immunohistochemistry may be also helpful in differential diagnosis between *usual* CA and CA with *atypia or sarcomatous transformation*. p16 expression is commonly negative in *usual* CA whilst is multifocal or diffuse in CA with *atypia or sarcomatous transformation* [9]. The p16 and p53 represent tumour suppressor genes involving in the regulation of molecular pathways that may play a role in the tumour progression in sarcomas: overexpression of p16 may participate in the molecular mechanisms underlying the atypical or sarcomatous transformation seen in some subset of CA [9].

Recently fluorescent *in situ* hybridization (FISH) [13] has shown a genetic relationship between CA, mammary-type myofibroblastoma and spindle cell lipoma. The same monoallelic or biallelic loss of retinoblastoma (RB) 1 (13q14), suggested a spectrum of one entity with morphological variations dependent on anatomic location. This argument doesn’t apply to solitary fibrous tumour as pubblished by Fritchie et al. [26] that shows the absence of monoallelic/biallelic RB1 loss by FISH, arguing against the concept the solitary fibrous tumour is genetically related to the other three entities.

RB is an important tumor suppressor protein that plays a crucial role in cell cycle progression [32]. RB gene is located at 13q14. Disruptions to the RB protein and to the pathway controlled by RB confer proliferative advantage to tumor cells [33]. In 13q14 gene is located also a tumor suppressor called Forkhead box protein O1 (FOXO1) [34]. FOXO1 is a transcription factor associated with apoptosis, cell cycle regulation, DNA repair and resistance to oxidative stress [34]. A loss of FOXO1 expression was recently associated with CA [35]. FOXO1 transcription factor induces an increased expression of manganese superoxide dismutase (MnSOD) resulting in an elimination of the reactive oxygen species (ROS) [36]. Hence, loss of FOXO1 expression is associated with a decreased expression of MnSOD and, as consequence, an increased intracellular ROS generation causing mutations in proto-oncogenes and tumor suppressor genes [37]. In fact, increased intracellular ROS induces p38 mitogen-activated protein (MAPK) pathway and may be linked to the tumorigenesis [36–38].

ROS can cause tumor development through cellular proliferation, tumor cell invasion, angiogenesis and cancer stem cell survival [37]. Also mammary and vaginal myofibroblastomas can present a monoallelic deletion of FOXO1 [31]. Hence, RB1 and FOXO1 loss of expression could be implicated in the pathogenesis of CA [13, 39]. RB1 and FOXO1 FISH analysis could be used to support CA diagnosis but the specificity is uncertain [13].

Also CA pathogenesis is still unclear, both sexual hormones and ROS has been proposed. Although human papillomavirus (HPV) E7 oncoprotein bind RB protein causing a loss of function [40], to our knowledge no role of infections in the pathogenesis of CA has been studied.

CA appears to behave in a benign fashion, since there is no report of tumours that progressed with metastasis and there is described only a recurrent case [12]. The primary and the recurrent CA consisted of a well circumscribed solid white mass of 4 and 6.5 cm respectively. The histology of both lesions didn’t consistently differ: the recurrent lesion showed foci of increased cellularity and decreased vascularity compared with the original specimen although there were no histological features to suggest malignancy and 33 months after the excision of the recurrent CA, there were no evidences of further local recurrence.

In the majority of the analysed cases, the surgical approach consists of a simple local excision or a “shelling out” and these treatments seem to be adequate also in case of atypia and/or sarcomatous transformation where the literature suggests a radical excision with free margins [9, 12]. To date, we are not aware of any cases of metastases of these tumours with atypia/sarcomatous features, suggesting that morphological atypical or sarcomatous aspects don’t necessarily confer an aggressive biologic behaviour to CA. Moreover, only 5 cases (5/18, 27.8 %) of re-excision because involved surgical margins has been reported but this method doesn’t represent an elective procedure because the tumour is usually treated with a local excision also when there are positive surgical margins, as showed in the remaining 13 cases (13/18, 72.2 %) with positive surgical margins, although no recurrences or metastasis have been reported. Therefore, considering that also cases of CA with positive margins don’t recur, no wide excision should be required. Given the relative sensitivity of the most frequently affected anatomic sites, there seems to be no justification for attempting to obtain a larger surgical margins [3].

## Conclusions

In summary, CA in women represents a distinct benign neoplasm with a broad anatomic distribution even if it is mainly localized in the vulvo-vaginal area. This lesion may exhibit some variations in its phenotypic features, as well as atypia and morphologic features of sarcomatous transformation but these characteristics seem not to predispose to a malignant fashion and recurrences. For these reasons, a treatment of simple local excision or a “shelling out” of the lesion appear to be adequate and effective to avoid recurrences and injuries to surrounding tissues.

## Abbreviations

CA: Cellular angiofibroma
AA: Aggressive angiomyxoma
AMF: Angiomyofibroblastoma
ER: Estrogen receptor
PR: Progesterone receptor
FISH: Fluorescent *in situ* hybridization
RB: Retinoblastoma
FOXO1: Forkhead box protein O1
MnSOD: Manganese superoxide dismutase
ROS: Reactive oxygen species
MAPK: Mitogen-activated protein
HPV: Human papillomavirus
